# *In Vitro* and *In Vivo *Anti-Inflammatory Properties of the Hydroethanolic Extract of the Roots of *Vernonia guineensis* (Asteraceae)

**DOI:** 10.1155/2023/7915367

**Published:** 2023-03-01

**Authors:** William Yousseu Nana, Justin Rodrigue Billong Mimb, Albert Donatien Atsamo, Eric Gonzal Tsafack, Stephanie Flore Djuichou Nguemnang, Zenab Linda Fagni Njoya, Vanessa Mba Matah Marthe, Yacine Karelle Madjo Kouam, Marius Mbiantcha, Gilbert Ateufack

**Affiliations:** ^1^Laboratory of Biology and Physiology of Animal Organisms, Department of Biology of Animal Organisms, Faculty of Science, The University of Douala Cameroon, P.O. Box 24157, Douala, Cameroon; ^2^Research Unit of Animal Physiology and Phytopharmacology, Faculty of Science, University of Dschang, Dschang, Cameroon; ^3^Laboratory of Animal Physiology, Faculty of Science, University of Yaounde I, Yaoundé, Cameroon

## Abstract

In traditional Cameroonian medicine, to relieve many inflammations, parts of *Vernonia guineensis*, are very widely used. This study considered the evaluation of acute toxicity and anti-inflammatory properties of the hydroethanolic extract of the roots of *Vernonia guineensis*. In an acute toxicity study, 250, 2500, and 5000 mg/kg were administered orally to mice in a single dose, and general behavior, adverse effects, and mortality were monitored. *In vitro* and *in vivo *anti-inflammatory tests were performed, and then histological, serum, hematological, and oxidative stress parameters have been evaluated. In an acute toxicity, all groups revealed neither mortality nor any significant alteration in behavior; only drowsiness, sedation, and lethargy were observed at 5000 mg/kg. For *in vitro* tests, the extract inhibited anti-inflammatory activity. In the formalin test, at 250 mg/kg, the extract inhibited edema with a percentage of 56.41% (4^th^ hour) in an acute treatment and 74.44% (10^th^ day). Joint edema was reduced by 67.24% (24^th^ hour) in a single treatment and by 74.25% (7^th^ day) in repeated treatment. The extract caused an increase in red blood cell, hemoglobin, and serum protein levels and reduced the white blood cells as well as the activities of alkaline phosphatase and alanine aminotransferase. The extract modulated oxidative stress parameters in the brain, spinal cord, liver, and kidneys. The extract protected the joint by reducing the bone and cartilage erosion. The present work highlights the anti-inflammatory, antioxidant, and antianemic properties of the hydroethanolic extract of the roots of *Vernonia guineensis*, which supports its empirical use in traditional medicine for the treatment of inflammatory pathologies.

## 1. Introduction

The inflammatory process, caused by tissue damage, infection, intense heat, or irritation, can be acute or chronic [1–3] with many phases of evolution (vascular, vasculo-exudative, cellular, and resolution). In addition, the state of chronic inflammation can lead to more complex pathologies such as rheumatoid arthritis and cancer [4]. Inflammatory diseases are disabling, affecting nearly 3% of people worldwide and 5.4% of people in Cameroon [5, 6]. During inflammation, we observe a dysfunction of the immune system [7] with the release of many proinflammatory mediators and the activation of many enzymes that promote the maintenance and aggravation of the inflammatory process [8]. Thus, the inflammatory process can occur in the absence of appropriate and effective treatments.

The Asteraceae, a large family of dicotyledonous plants, represents the second family of the plant world since it includes nearly 23,500 species distributed in approximately 1,600 genera [9]; its essential representatives develop particularly in dry regions, outside of competition from tropical rainforest trees [10]. This family particularly includes herbaceous plants, some trees, shrubs, or lianas [9]. They are perennial plants with simple or branched taproot roots, sometimes tuberous. It can be annuals (Helianthus, Tagetes, marigolds, and lettuces), shrubs (*Vernonia arborea* and *Leucomeris*), hydrophytes (Bidens), helophytes (*Sphaeranthus indicus*), xerophytes (Proustia), or climbing lianas (Mikania). *Vernonia guineensis* (*V. guineensis*) which belongs to the vast Asteraceae family is a plant consisting of a short, underground, and perennial stem formed of tuberous roots, the stems, covered with abundant gray hairs, are generally branched, erect, and do not exceed 1 meter. The denticulated, oval leaves, presenting several shapes, have a shiny/smooth side, and the other is covered with a thick hairy felting [11]. This plant is distributed from the region of Mali to West Cameroon, then through Central Africa from Cameroon to Sudan [12]. Traditionally, all parts of *V. guineensis* are used and prepared in different ways to treat many diseases in West Africa and Cameroon [13, 14]. Indeed, *V. guineensis* is used in Côte d'Ivoire against arterial hypertension, typhoid fever, malaria, childhood diseases, diabetes, and epilepsy [15], its roots are macerated to the treatment of strangulated hernia [16]. In Congo-Zaire, its leaves are used to treat wounds and arthritis, and its roots treat helminthiasis [17]; while in Angola, its roots treat hernia, childhood illnesses, abdominal pain, and wounds [18]. In South and West Cameroon, *V. guineensis* is used as an aphrodisiac, anthelmintic, and antidote and also to treat epilepsy, sinusitis, abnormal menstruation, malaria, inflammatory conditions, and jaundice [19–22]. Previous studies conducted on *V. guineensis* showed that this plant was antimicrobial, vermifuge, and antiplasmodial [23–25], it was also anticancer and antiangiogenic [26–28]. The study of Toyang et al. [29] shows that acute administration of an extract from *V. guineensis* did not produce toxic effects in rats and that the absence of acute toxicity at the highest concentration tested indicates that this plant is safe at concentrations ≤4000 mg/kg. At the same, Nnanga et al. [30] show that administration of *V. guineensis* at 400 mg/kg for 14 consecutive days exhibited hepato-protective activity and improved glomerular filtration. Furthermore, compounds such as vernodalin and vernolepin [31], vernoguinosterol and vernoguinoside [32], vernopicrin-1, vernomelitensin, and pentaisovaleryl sucrose [26, 29], and several triterpenes and steroids [25] have been isolated from this plant. The work of Collins Njonte Wouamba et al. [33] made it possible to isolate several secondary metabolites from this plant, namely quercetin, vernopicrin, luteolin, *β*‐amyrin, vernomelitensin, lupeol, *β*‐carotene, oleanolic acid, betulinic acid, a mixture of *β*‐sitosterol and stigmasterol, ursolic acid, 2,3-dihydroxypropyl heptacosanoate, *β*-sitosterol-3-O-*β*-D-glucoside, heptatriacontan-1-ol, pentacosanoic acid, tritriacontan-1-ol, and docosan-1-ol. Several of these compounds have already proven their pharmacological properties in many pathologies including inflammatory pathologies. Our preliminary work carried out *in vitro* showed that the hydroethanolic extract of the roots of *V. guineensis* was able to inhibit the activities of COX, 5-LOX, inhibit protein denaturation, and proteinase; this is why this study evaluated the anti-inflammatory effect of this extract on inflammation models (acute and chronic).

## 2. Material and Methods

### 2.1. Plant Material

*V. guineensis*, whole plant, harvested (February 2019, locality of Bangangté, West Cameroon), was authenticated in the national herbarium of Cameroon in Yaoundé by comparison with the existing specimen bearing the name of LETOUZEY under the number 4869/SRFK. The roots harvested after identification were washed with water, cut into fine pieces, dried (open air sheltered from the sun), and crushed (moulinette) to give a fine powder which made it possible to prepare the extract. Eight hundred (800) g of powder were soaked in 6 liters of ethanol/distilled water (70/30) mixture. The mixture was macerated several times for 48 hours and then filtered (Whatman No. 3 paper). The filtrate obtained was first evaporated in a rotary evaporator (60°C), then in an oven (40°C), this made it possible to obtain 59.5 g of dry mass representing the hydroethanolic extract for a yield of 7.44%.

### 2.2. Quantitative Phytochemistry of the Hydroethanolic Extract *V. guineensis*

#### 2.2.1. Dosage of Total Phenols

Total phenols were assayed according to the protocol using the Folin–Ciocalteu reagent [34]. Gallic acid (0.01, 0.02, 0.03, 0.04, and 0.05 mg/ml) was used as standard and the results were expressed in mg/g of gallic acid equivalents (GAE). Gallic acid and whole extract (0.1 and 1 mg/ml) were prepared using the methanol. A mixture of the sample (0.5 ml), Folin–Ciocalteu's reagent (2.5 ml) diluted 10 times (7.5 mg of gallic acid (2.5 ml), and 7.5% of carbonate sodium (2 ml)) were introduced into the tubes which were covered and left 30 minutes (room temperature), andthen the absorbance (760 nm, spectrophotometer) was read.

#### 2.2.2. Dosage of Total Flavonoids

The aluminum chloride colorimetric protocol described by Chang et al. [35] served as support to evaluate the total flavonoid contents in the hydroethanolic extract of *V. guineensis*. A mixture of extract (100 *µ*l and 2 mg/ml), aluminum chloride (50 *µ*l and 1.2%), and potassium acetate (50 *µ*l and 120 mM) introduced into a tube was incubated (room temperature, 30 minutes) and the absorbance was read (415 nm, spectrophotometer). Various concentrations of quercetin (0.015 to 2 mg/ml) were used to plot the standard curve which allowed the calculation of the total flavonoid content in the extract.

#### 2.2.3. Dosage of Total Tannins

The Folin–Ciocalteu method described by Govindappa et al. [36] was used to determine the total tannin content in Vernonia guineensis extract. The mixture consisting of extract (100 *µ*l, 2 mg/ml), Folin–Ciocalteu reagent (500 *µ*l, diluted 10 times in water), sodium carbonate (1000 *µ*l, 35%), and distilled water (8.4 ml) was shaken, incubated (room temperature, 30 minutes), and the absorbance (700 nm, spectrophotometer) was read. The various concentrations of tannic acid (100 to 500 *µ*g/ml) were used to draw the standard curve which made it possible to calculate the content of total tannins in the extract.

### 2.3. *In Vitro *Anti-Inflammatory Activity

#### 2.3.1. Lymphocyte Culture, Assay of Cyclooxygenase, 5-Lipoxygenase, and Inhibition of Protein Denaturation

A culture of human peripheral lymphocytes ((RPMI 1640 (HIMEDIA), fetal bovine serum, streptomycin, phytohemagglutinin (HIMEDIA), and penicillin) was filtered (cellulose acetate, 0.2 *µ*m, Sartorios) and plasma was added (1 × 106 cells/ml), then the culture was incubated (72 h). Thereafter, the lipopolysaccharide (1 *µ*l) was added followed by a new incubation (24 h), then the extract (50, 100, 200, and 400 *µ*g/ml) or diclofenac (50, 100, 200, and 400 *µ*g/ml) or ibuprofen (50, 100, 200, and 400 *µ*g/ml) was added, after a new incubation (24 h) and centrifugation (10 minutes, 6000 rpm), the supernatant was removed and the lysis buffer (50 *µ*l) was added and the mixture was again centrifuged [37].

For COX, trisHCl buffer, glutathione, hemoglobin, enzyme, arachidonic acid, and 0.2 ml TCA (10% in 1 N HCl) were mixed and incubated (37°C, 20 minutes), 0.2 ml of TBA was added and then we heated (boiling bath, 20 minutes), cooled, and centrifuged (3 minutes, 1000 rpm), and the activity (632 nm) of COX was read in the supernatant [38].

For 5-lipoxygenase, linoleic acid (70 mg), Tween 20 (4 ml), 0.5 N sodium hydroxide, and oxygen-free water were mixed to give 25 ml of a solution that was divided into portions (0.5 ml), rinsed with nitrogen, frozen and placed in a cuvette (quartz, 25°C). The mixture of sodium linoleate (0.2 ml), Tris buffer (2.75 ml, pH 7.4), and enzyme (50 ml) constituted the control. The optical density read at 234 nm, made it possible to determine the percentage inhibition of 5-LOX activity [37, 38].

For protein denaturation, distilled water or diclofenac (50, 100, 200, and 400 *µ*g/ml) or extract (50, 100, 200, and 400 *µ*g/ml) was mixed with bovine serum (5%, 1 ml), incubated (27°C, 15 minutes), placed for 10 minutes (70°C), and allowed to cool, optical density was read (660 nm) and percent inhibition of protein denaturation was calculated [39].

#### 2.3.2. Evaluation of the Effect of the Extract on Proteinase

A mixture of trypsin (2 ml, 6%), tris-HCl buffer (1 ml, pH 7.4, 20 Mm), extract (1 ml, 50, 100, 200, and 400 *µ*g/ml), or diclofenac (1 ml; 50, 100, 200, and 400 *µ*g/ml) were incubated (5 minutes, 37°C), then casein (1 ml, 0.8%) was added followed by a new incubation (20 minutes, 37°C); subsequently perchloric acid (70%) was introduced into the mixture and the optical density was read at 120 nm against the blank and the percentage inhibition of proteinase activity was calculated [40].

#### 2.3.3. Evaluation of the Effect of the Extract on the Stabilization of Erythrocyte Membranes

Rat blood, mixed with EDTA (ethylenediaminetetraacetic acid), was centrifuged (3000 rpm, 10 minutes, 25°C); after washing the supernatant (three times), the blood was reconstituted under suspension form (10%; v/v, physiological serum) [40].

A rich mixture (2 ml) of 1 ml of extract (50, 100, 200, and 400 *µ*g/ml) or diclofenac (50, 100, 200, and 400 *µ*g/ml), 1 ml of red blood cells (10%) and saline solution, was incubated (56°C, 30 minutes), cooled (running water), and then centrifuged (5 minutes 2500 rpm) followed by the reading of the absorbance (560 nm) in the supernatant and calculation of the percentage of membrane stabilizing activity [40].

### 2.4. *In Vivo* Tests

#### 2.4.1. Animal Material

The animals used in the present study were mice (*Mus musculus*, female and male, 3 months, 23–28 g) and rats (*Wistar*, Ratu sratus, female and male, 3 months, 150–180 g), bred (room temperature, natural day/night cycle, drinking water and food) in the animal facility of the Faculty of Science (University of Dschang), housed in cages at a rate of 5 animals for each cage. The laboratory committee (Laboratory of Animal Physiology and Phytopharmacology, Department of Animal Biology, University of Dschang-Cameroon) approved all the protocols used, based on the ethical guidelines (laboratory animal use) described in the European Community guidelines (EEC Directive 86/609/EEC, 24^th^ November 1986) [41].

#### 2.4.2. Acute Toxicity Study

This study was carried out according to the OECD (Organization for Economic Cooperation and Development) protocol described by Walum [42]. After fasting for 12 hours with free access to drinking water, 24 mice were weighed, divided according to their mass into 4 groups of 6 mice each, and treated as follows: group 1 consisting of mice treated with distilled water; groups 2, 3, and 4 consisting of mice treated with the hydroethanolic extract of *Vernonia guineensis* at the respective doses of 250, 2500, and 5000 mg/kg. After the oral administration of the different solutions, the food was suspended for the first 4 hours. The animals were observed for the first 4 hours postgavage and then for a period of 7 days to look for possible toxic effects [43]. In addition to mortality, animal behavior, body weight, food intake, urination, water intake, convulsions, respiration, tremors, constipations, temperature, color change of skin, locomotion, eye color change, and many other parameters were observed during these periods [43, 44].

### 2.5. Anti-Inflammatory Activity

#### 2.5.1. Distribution of Animals

For all tests, animals were allocated and treated as follows: after fasting for 24 hours, animals in group 1 (neutral control) received distilled water, animals in group 2 (negative control) received distilled water, animals in group 3 (positive control) received diclofenac (5 mg/kg), and animals of groups 4, 5, and 6 (treated) received the hydroethanolic extract of *V. guineensis* (62.5, 125 and 250 mg/kg).

#### 2.5.2. Formalin-Induced Acute Inflammation

The paw diameter of each animal was measured before any treatment using a caliper, representing the baseline value (0 h); then, each animal received orally (1 mL/100 g, b.w.), using a gavage probe, the corresponding treatment. One hour after this treatment, each rat received (exception for the animals in the neutral control group) an injection of 2.5% formalin under the plantar aponeurosis of the left hind paw using a 1 mL syringe, then the diameter of the paw was measured 1 h, 2 h, 4 h, 8 h, and 24 h after formalin injection [41]. The extent of edema was assessed by determining the percentage increase in the volume of the sole of the paw [4].

(1) $$\begin{array}{c} Increase\;of\;Paw\;Volume\left(IPV\right)=\frac{Paw\;\;Volume\;at\;time\;T-Initial\;Paw\;Volume}{Initial\;Paw\;Volume}X100\text{,}\\ Percentage\;Inhibition=\frac{\left(IPV\right)control–\left(IPV\right)treated}{\left(IPV\right)control}X100\text{.} \end{array}$$

#### 2.5.3. Formalin-Induced Chronic Inflammation

For this test, the animals from the previous test (acute inflammation) were kept and received a second injection (2.5%, formalin) on the third day after the start of the first injection. Then, these animals received different treatments every day for 10 days consecutively; then, the percentages of inhibition were calculated as previously described.

#### 2.5.4. Carrageenan-Induced Joint Inflammation

For this test, other male and female rats were selected based on their body mass, divided, and treated as previously described. Subsequently, the protocol described by Bang et al. [45] was used. The treatments were administered orally due to 1 mL/100 g of body mass and 1 hour after administration of each treatment, and carrageenan solution (0.1 mL, 3%) was injected into the ankle joint of the left hind paw of each rat (except the neutral control group). The diameter of the joint was measured using a caliper 0 h before any treatment, then 1 h, 2 h, 4 h, 6 h, and 24 h, then from day 2 to day 10 after injection of carrageenan. Treatments were also given daily from day 2 through day 10. The extent of edema was assessed as previously described in the formalin test.

On day 11, animals were anesthetized with thiopental (50 mg/ml, i.p.), blood was drawn (catheterization and abdominal artery), and introduced into tubes containing EDTA (ethylene- diamine-tetra-acetic) for the evaluation of red blood cells, hemoglobin, white blood cells, hematocrit, and platelets using an automatic hematological analyzer (Sysmex pocH-100i); then in other tubes without anticoagulant which were centrifuged cold (4°C) at 3000 rpm for 15 minutes using a centrifuge (Eppendorf 5804R, Hamburg), and the supernatant obtained was collected (Eppendorf tubes) for alanine aminotransferase (ALT), total protein, aspartate aminotransferase (AST), creatinine, and alkaline phosphatase (ALP) assays. Subsequently, the organs (brain, spinal cord, kidneys, liver, and spleen) were removed, rinsed in 0.9% NaCl, dried with paper towels, and then weighed using a sensitive balance. The brain, spinal cord, liver, and kidney were ground in PBS due to 0.1 g organ per 1 ml buffer. After grinding, the different homogenates were centrifuged (4°C, 3000 rpms/min, 15 minutes), using a centrifuge (Eppendorf 5804R, Hamburg), and the supernatants were collected in tubes with Eppendorf and stored at −20°C for the determination of catalase (CAT), malondialdehyde (MDA), glutathione (GSH), nitric oxide (NO), and superoxide dismutase (SOD). Subsequently, the ankle joint was also removed and preserved in 10% formalin buffered with PBS for the production of histological sections.

### 2.6. Statistical Analyzes of Data

The results obtained in each series of experiments were expressed as the mean ± standard error of the mean (SEM). The averages were analyzed first by the one-way analysis of variance test (ANOVA) (mass of the organs, biochemical, and hematological parameters) then two-way (paw diameter, joint diameter) followed by Tukey's and Bonferroni's post-test, respectively. The differences were significant at the probability thresholds *P* < 0.05. The calculation of the mean of each series of values as well as the statistical analysis were carried out using the software Graph Pad (Instant Biostatistic) version 3.0.

## 3. Results

### 3.1. Quantitative Analysis of Extracts

The quantitative phytochemistry carried out on the hydroethanolic extract of *V. guineensis* shows that the extract contains flavonoids, phenols, and tannins at respective proportion as 138.31 mg equivalent of quercetin/g, 223.21 mg/g catechin, and 119.29 mg equivalent of tannic acid/g (Table 1).

### 3.2. *In Vitro *Anti-Inflammatory Activities of Hydroethanolic Extract of *V. guineensis*

Table 2 shows the effects of hydroethanolic extract of *V. guineensis* on cyclooxygenase, 5-lipoxygenase, proteins denaturation, proteinase, membrane stabilisation, and no production. It emerges from this table that, at a concentration of 400 *µ*g/ml, the extract inhibits cyclooxygenase, 5-lipoxygenase, proteins denaturation, proteinase, and membrane stabilization with percentages of 80.19%, 64.66%, 79.02%, 83.88%, and 80.57%, respectively (Table 2).

### 3.3. *In Vivo* Test

#### 3.3.1. Acute Toxicity

The dose limits used for acute oral toxicity studies were selected in accordance with OECD guidelines. At doses of 250, 2500, and 5000 mg/kg, no treatment-related toxic symptoms or mortality were observed. Alertness, touch response, grooming, restlessness, pain response, flare response, tremors, righting reflux, convulsions, grasping, corneal reflex, pinna reflex, writhing, pupils, urination, salivation, skin color, lacrimation, water consumption, food intake, and mortality did not change in extract-treated animals compared to treated with distilled water (Table 3). Nevertheless, signs of lethargy, sedation, and drowsiness were observed in some animals at the 5000 mg/kg dose in the first 4 hours. No case of death having been observed up to the dose of 5000 mg/kg, and the LD_50_ is therefore considered to be greater than 5000 mg/kg.

### 3.4. Anti-Inflammatory Properties

#### 3.4.1. Effects of the Hydroethanolic Extract of *V. guineensis* on Acute and Chronic Edema Induced by Formalin

**Figure 1:**
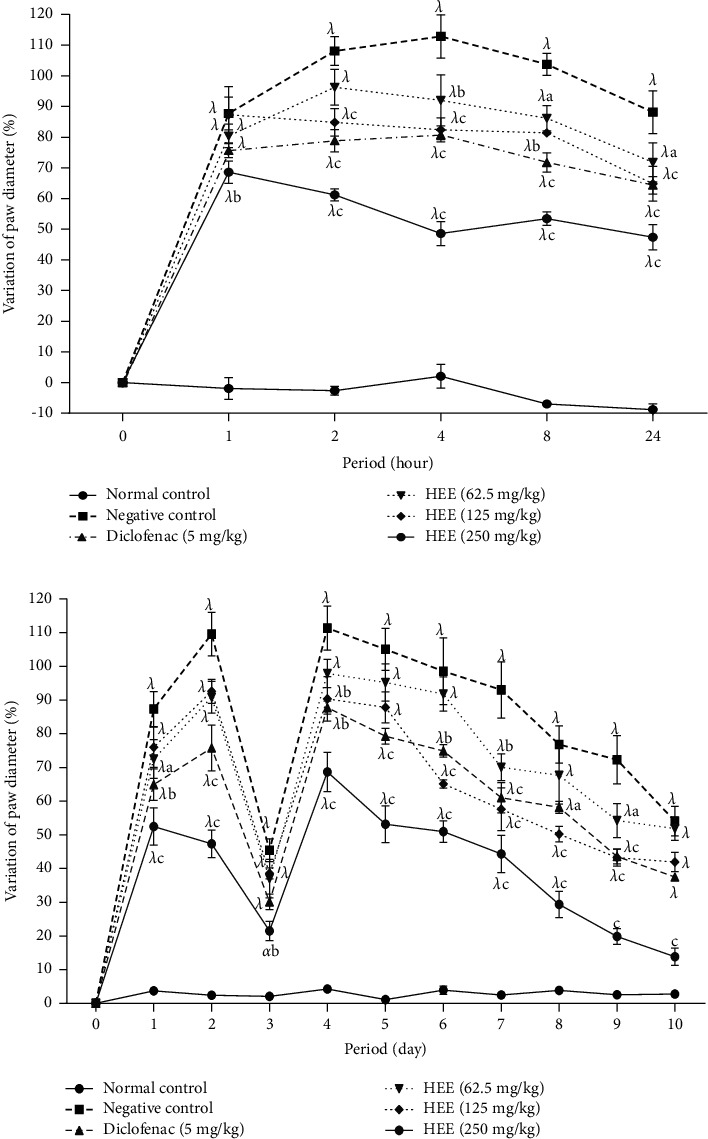
Effect of the hydroethanolic extract of *V. guineensis* on the thickness of the paw after the induction of formalin in (a) acute and (b) chronic inflammation. Each value represents the mean ± SEM of 5 animals; ^a^*p* < 0.05, ^b^*p* < 0.01, and ^c^*p* < 0.001 are significantly different from negative control; ^*α*^*p* < 0.05 and ^*λ*^*p* < 0.001 are statistically significant compared to the neutral control.

Figure 1(a) presents the effects of the hydroethanolic extract of *V. guineensis* on the variation in the thickness of the paw after injection of formalin (2.5%) in rats. It is apparent from this figure that formalin injection resulted in a significant (*P* < 0.001) increase in paw thickness in all animals from the first hour compared to animals in the neutral control group. Thus, this increase was maximal in the negative control at the 4th hour, i.e., 112.08% (*P* < 0.001) increase compared to its baseline value. However, administration of the extract at doses of 62.5, 125, and 250 mg/kg significantly (*p* < 0.05, *p* < 0.01, and *p* < 0.001) decreased paw thickness compared to animals given distilled water, with maximal inhibition percentages 18.62% (24^th^ hour), 26.99% (4^th^ hour), and 56.41% (4^th^ hour), respectively, at 62.5 mg/kg, 125 mg/kg, and 250 mg/kg. It emerges from Figure 1(b) that the hydro-ethanolic extract of *V. guineensis* at a dose of 250 mg/kg, as well as the diclofenac (5 mg/kg) decreased significantly (*p* < 0.05, *p* < 0.01, and *p* < 0.001) the thickness of the paw on the second day after injection of the formalin compared to the negative control group. Paw thickness fell on day 3 in all groups before increasing on day 4 following a second injection of formalin on day 3. A significant decrease (*p* < 0.05, *p* < 0.01, and *p* < 0.001) in all groups treated with the extract and the reference substance compared to the negative control group was observed after the second injection. The maximum inhibition of 74.44% was observed with extract (50 mg/kg) on the tenth day, while diclofenac produced a maximum effect of 30.70% on the 9^th^ day.

#### 3.4.2. Effect of the Hydroethanolic Extract of *V. guineensis* on the Diameter of the Joint after Injection of Carrageenan

**Figure 2:**
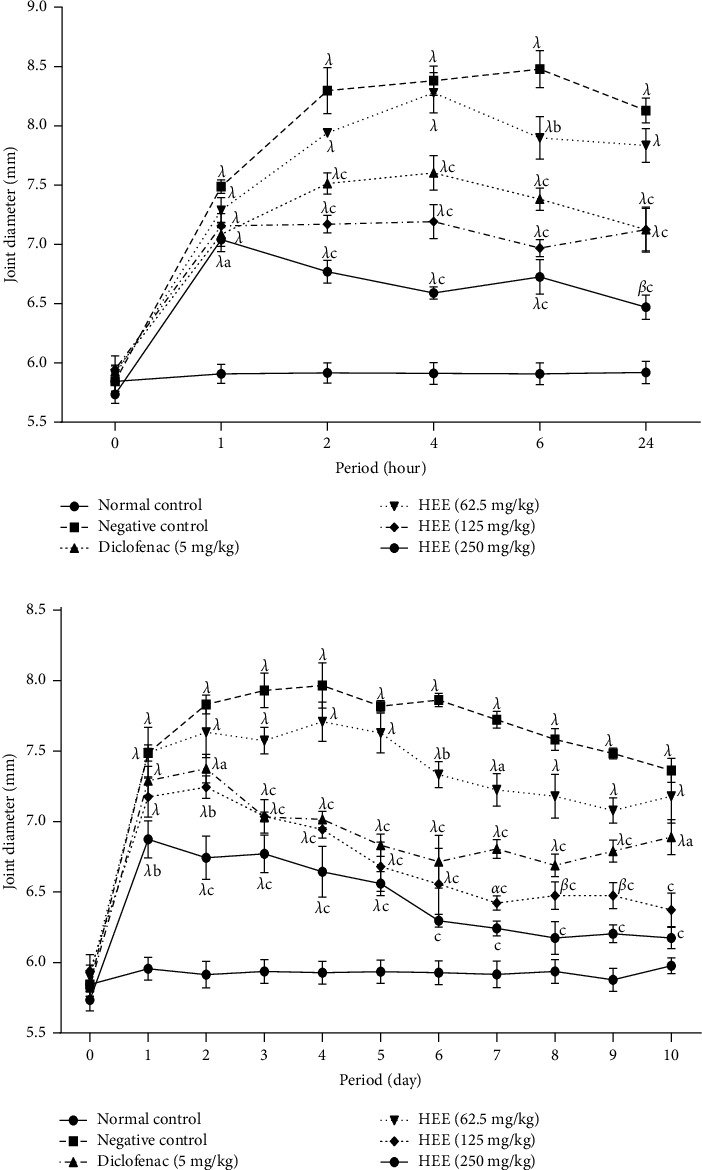
Effect of the hydroethanolic extract of *V. guineensis* in (a) single and (b) continuous treatments on the thickness of the joint after the injection of carrageenan. Each value represents the mean ± SEM of 5 animals; ^a^*p* < 0.05, ^b^*p* < 0.01, and ^c^*p* < 0.001 are significantly different from negative control; ^*α*^*p* < 0.05, ^*β*^*p* < 0.01, and ^*λ*^*p* < 0.001 are statistically significant compared to the neutral control.

Figure 2 presents the effects of a hydroethanolic extract of *V. guineensis* on carrageenan-induced joint edema. This figure shows that the injection of carrageenan at the level of the joint leads to a significant increase (*p* < 0.001) in the diameter of the joint in all the animals compared to the neutral control group after one hour. In the negative control, this increase went from 5.85 mm (baseline value) to 8.48 mm at the 6th hour (maximum value). From the second to the twenty-fourth hour, the administration of the hydroethanolic extract of *V. guineensis* at doses of 125 and 250 mg/kg, as well as diclofenac at a dose of 5 mg/kg decreased significantly (*p* < 0.001) joint thickness compared to the group that received distilled water as a treatment. Maximum inhibitions of 61.42% (6th hour) and 67.24% (24^th^ hour) were obtained at 125 and 250 mg/kg. Diclofenac induced 48.44% inhibition at the 24^th^ hour (Figure 2(a)). It appears from Figure 2(b) that the injection of carrageenan in rats caused a significant increase (*p* < 0.05, *p* < 0.01, and *p* < 0.001) and sustained for 10 days in the thickness of the joint compared to animals that did not receive carrageenan. The administration of the extract at 125 and 250 mg/kg as well as the reference substance significantly reduced (*p* < 0.05, *p* < 0.01, and *p* < 0.001) the thickness of the joint of the day 2 to end of treatment compared to animals given distilled water as treatment. At doses of 62.5 mg/kg, 125 mg/kg, and 250 mg/kg, the maximum effects observed were 29.85% (7^th^ day), 62.57% (7^th^ day), and 74.25% (7^th^ day). The maximum effect observed in animals treated with diclofenac (5 mg/kg) was 55.93% on day 6.

#### 3.4.3. Effects of Hydroethanolic Extract of *V. guineensis* on Organ Mass

**Figure 3:**
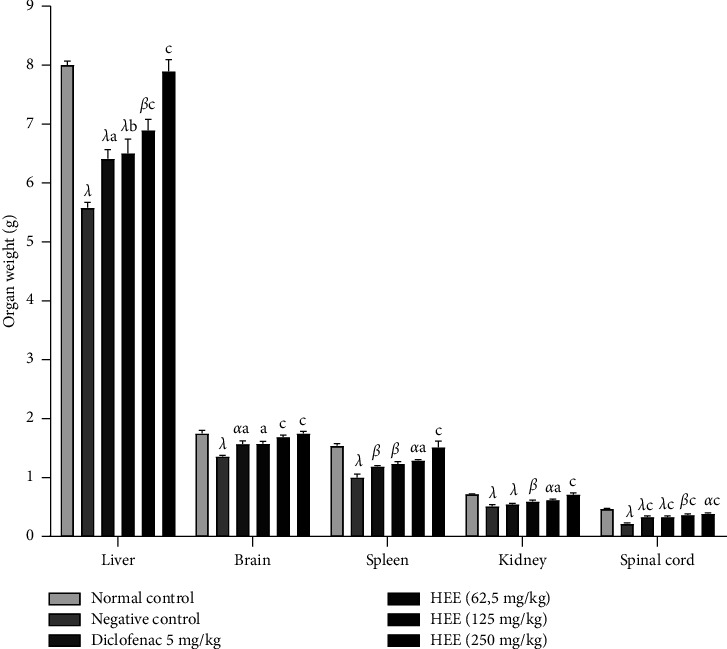
Effect of the hydroethanolic extract of *V. guineensis* on the mass of some organs after injection of carrageenan. Each histogram represents the mean ± SEM, *n* = 5; ^a^*p* < 0.05, ^b^*p* < 0.01, and ^c^*p* < 0.001 are significantly different from negative control; ^*α*^*p* < 0.05, ^*β*^*p* < 0.01, and ^*λ*^*p* < 0.001 are statistically significant compared to the neutral control.

Figure 3 shows the effect of the hydroethanolic extract of *V. guineensis* on the mass of the liver, brain, spleen, kidneys, and spinal cord after ten days of treatment. It is observed in this figure that the administration of the hydroethanolic extract of *V. guineensis* at 62.5; 125 and 250 mg/kg resulted in a significant (*p* < 0.05, *p* < 0.01, and *p* < 0.001) increase in liver mass compared to the negative control group. Regarding the brain and spinal cord, *V. guineensis* extract at all doses as well as the reference drug increased significantly (*p* < 0.05, *p* < 0.01, and *p* < 0.001) brain and spinal cord mass compared to the negative control group. The hydroethanolic extract of *V. guineensis* also significantly (*p* < 0.001) increased spleen and kidney masses compared to the negative control group.

#### 3.4.4. Effect of the Hydroethanolic Extract of *V. guineensis* on Some Hematological Parameters

Table 4, which shows the hematological variations after 10 days of treatment with the hydroethanolic extract of *V. guineensis*, shows that the levels of white blood cells (*p* < 0.001) and platelets (*p* < 0.01) increase significantly, while the levels of red blood cells (*p* < 0.001) and hemoglobin (*p* < 0.001) decrease significantly in the animals of the negative control group compared to the animals of the neutral control group. In the animals treated with 125 and 250 mg/kg of the hydroethanolic extract of *V. guineensis*, the levels of the hematological parameters evaluated tended to return to normal, with a significant decrease (*p* < 0.001) in the levels of white blood cells and blood platelets and a significant increase (*p* < 0.001) in red blood cells and hemoglobin compared to those of animals in the negative control group.

#### 3.4.5. Effect of the Hydroethanolic Extract of *V. guineensis* on Some Serum Biochemical Parameters

Table 5 shows the effect of *V. guineensis* extract on ALT, AST, alkaline phosphatase activities, creatinine, and total protein levels. It appears from this table that the injection of formalin in rats led to a significant increase (*p* < 0.05 and *p* < 0.01) in the activities of ALT and PAL, and a decrease in total protein levels in the animals of the negative control group compared to the animals of the neutral control group. Administration of *V. guineensis* extract at 125 and 250 mg/kg significantly decreased (*p* < 0.001) the activities of these enzymes and significantly increased (*p* < 0.01) protein levels compared to the negative control group. Regarding to AST activity and creatinine level, no significant variation is observed in animals treated with *V. guineensis* extract and the reference substance compared to the neutral control group and to the animals of the negative control group.

#### 3.4.6. Effect of the Hydroethanolic Extract of *V. guineensis* on Some Parameters of Oxidative Stress

**Figure 4:**
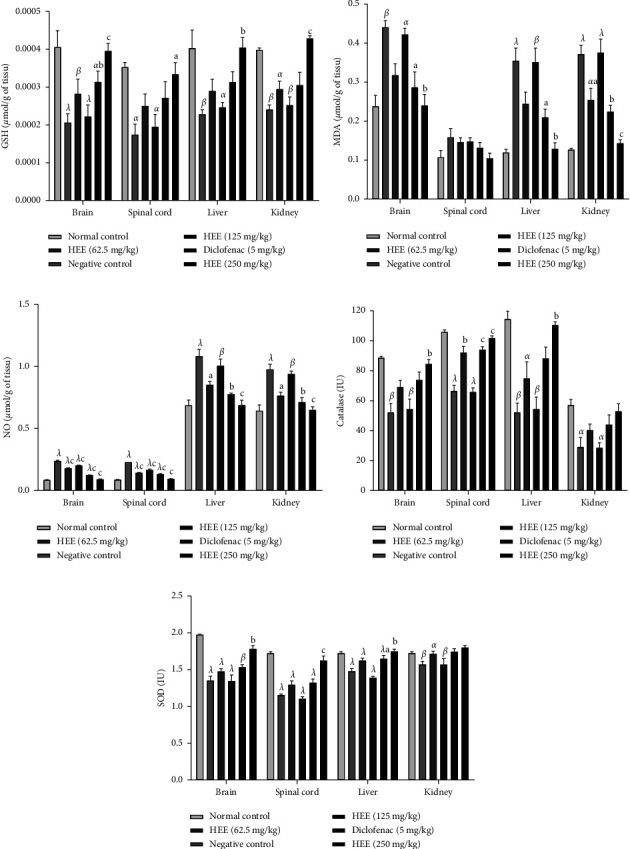
Effect of hydroethanolic extract of *V. guineensis* on glutathione, MDA, nitric oxide levels, catalase, and SOD activities in the brain, spinal cord, liver, and kidneys. Each histogram represents the mean ± SEM, *n* = 5; ^a^*p* < 0.05, ^b^*p* < 0.01, and ^c^*p* < 0.001 are significanly different compared to the negative control; ^*α*^*p* < 0.05, ^*β*^*p* < 0.01, and ^*λ*^*p* < 0.001 are statistically significant compared to the neutral control.

Figure 4 shows the effect of the hydroethanolic extract of *V. guineensis* on the levels of glutathione, MDA, catalase, NO, and SOD in the brain, spinal cord, liver, and kidney of rats 10 days after injection of carrageenan. This figure shows that the hydroethanolic extract of *V. guineensis* at 125 and 250 mg/kg significantly increased (*p* < 0.01, *p* < 0.001) the level of glutathione in the brain in comparison to animals in the negative control group. Regarding the spinal cord, liver, and kidney, the extract at 250 mg/kg caused a significant increase (*p* < 0.05, *p* < 0.01, and *p* < 0.001) in glutathione in comparison to the negative control group (Figure 4(a)). The administration of the hydroethanolic extract of *V. guineensis* (125 and 250 mg/kg) significantly reduced (*p* < 0.05 and *p* < 0.01) the level of MDA in the brain and liver in comparison to the negative control group. With regard to the kidney, the extract at 125 and 250 mg/kg, as well as the reference substance led to a significant reduction (*p* < 0.05, *p* < 0.01, *p* < 0.001) in the rate of MDA compared to animals that did not receive treatment (Figure 4(b)). In the liver and kidneys, administration of the extract (125 and 250 mg/kg), as well as the reference substance, significantly reduced (*p* < 0.05, *p* < 0.01, and *p* < 0.001) the NO rate compared to the negative control group (Figure 4(c)). Administration of *V. guineensis* extract (250 mg/kg) resulted in a significant (*p* < 0.01) increase in catalase in the brain and liver. Similarly, in the spinal cord, administration of this extract (125 and 250 mg/kg), as well as the reference substance, caused a significant increase (*p* < 0.01, *p* < 0.001) in catalase (Figure 4(d)). In the liver, *V. guineensis* extract at 125 and 250 mg/kg significantly (*p* < 0.05 and *p* < 0.01) increased SOD activity (Figure 4(e)).

#### 3.4.7. Effect of the Extract on Joint Histology

**Figure 5:**
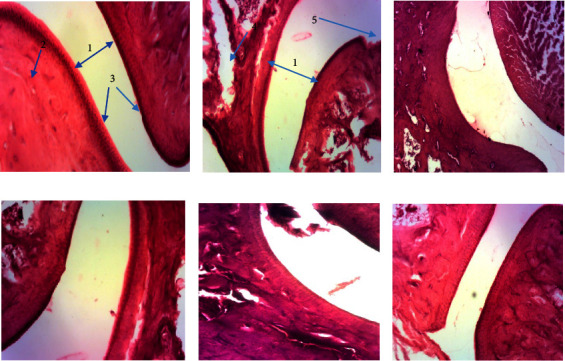
Micrograph of the tibiotarsal joint (hematoxylin-eosin X 100): (a) neutral witness, (b) negative control (distilled water), (c) diclofenac (5 mg/kg), (d) extract (62.5 mg/kg), (e) extract (125 mg/kg), and (f) extract (250 mg/kg). 1: joint space; 2: bone; 3: articular cartilage; 4: bone erosion; 5: cartilage erosion.

Figure 5 presents the histological of the joints after 10 days following an injection of carrageenan. It is observed in this figure that the animals of the neutral control group present normal joints with reduced joint space, no bone erosion, and intact cartilage (Figure 5(a)). Animals in the negative control group exhibit a joint with wide joint space, and erosion of bone and cartilage (Figure 5(b)). However, the animals having received the 250 mg/kg dose of the hydroethanolic extract of *V. guineensis* present a joint with little bone and cartilage erosion, and the joint space is reduced. Nevertheless, the animals having received the extracts (62.5 and 125 mg/kg) and diclofenac (5 mg/kg) presents a joint with a wide joint space.

## 4. Discussion

This study aimed to evaluate the acute toxicity and anti-inflammatory properties (*in vitro* and *in vivo*) of the hydroethanolic extract of the roots of *V. guineensis* in rats. The results of the present study reveal the hydroethanolic extract of the roots of *V. guineensis* in single administration as in repeated administration inhibited in rats; acute or chronic inflammation induced, respectively, by formalin or carrageenan. This work demonstrates for the first time the anti-inflammatory properties of *V. guineensis*.

The World Health Organization (WHO) recognizes medicinal plants as the main source of pharmaceutical products and recommends that they should be studied to better understand not only their medicinal properties and their efficacy but also their safety [46]. In this study, up to a dose of 5000 mg/kg, no signs of toxicity or mortality in animals were observed. These observations are in agreement with the work of Toyang et al. [29] who studied the acute toxicity of the aqueous extract of this same plant up to a dose of 5000 mg/kg reported no change in behavior and no mortality. According to the OECD [47], any substance or pharmaceutical product with an LD_50_ in oral treatment greater than 5000 mg/kg can be considered safe and nontoxic. This suggests that the hydroethanolic extract of *Vernonia guineensis* is practically nontoxic.

Injection of formalin into the paw of rats causes acute inflammation resulting in tissue damage due to the production of endogenous mediators such as histamine, serotonin, prostaglandins, and bradykinins [48]. These mediators cause an acute inflammatory reaction characterized by vasodilation, leukocyte infiltration in the tissues, and the formation of edema [49]. Formalin-induced inflammation was significantly inhibited by the hydroethanolic extract of the roots of *V. guineensis*. This activity was significant at high doses of extract from the 2^nd^ hour and maintained until the 24^th^ hour in a single treatment. The extract was also tested in the chronic model of formalin-induced inflammation. For it is well known that inhibition of formalin-induced edema in rats is one of the most suitable procedures for demonstrating antiarthritic and anti-inflammatory agents, as this model is closely similar to the human arthritis [50]. It is established that this type of inflammation is characteristic of chronic inflammatory reactions and can serve as a model for subchronic and chronic inflammation tests in order to evaluate the antiarthritic and antiproliferative activity of certain substances since it associates the proliferative phase of inflammation [50]. Morphologically, chronic inflammation is the presence of lymphocytes, plasma cells, and macrophages in the tissues [51]. During chronic inflammation, there is the release of certain mediators such as proinflammatory cytokines (IL and TNF-alpha), colony stimulating factors (CSF), interferons and platelet derived growth factors (PDGFs) [52–54], and free radicals such as NO [55]. The results also show that the hydroethanolic extract of the roots of *V. guineensis* significantly and in a dose-dependent manner inhibited the chronic inflammation induced by formalin. The effects of the extract in acute and chronic models of formalin-induced inflammation suggest that this plant possesses chemical compounds with inhibitory activities on the production of proinflammatory mediators (histamine, serotonin, prostaglandins, and bradykinins) or alternatively cyclo-oxygenase; in addition, would also possess antiarthritic and antiproliferative activity. This is more likely since Johnson et al. [56] and Mendis et al. [57], respectively, showed the antiproliferative properties of *Vernonia amygdalina*, a plant of the same genus as *V. guineensis*, and of a sesquiterpene lactone, a compound already isolated from *V. guineensis* [27].

It is known that several anti-inflammatory substances have an effect on certain enzyme systems such as transaminases [4]. The inhibition of transaminases observed with anti-inflammatory substances could influence the continued formation of polypeptide-kinins during the anti-inflammatory process [48]. The inhibitory effect of these anti-inflammatory substances may also be responsible for the decrease in the synthesis of mucopolysaccharides, which are mainly involved in the proliferative phase of inflammation [58]. The activity of transaminases (ASAT and ALAT), protein levels, and serum creatinine are excellent anti-inflammatory measures of a drug target. The role of transaminases in the formation of biologically active chemical mediators such as bradykinins during acute or chronic inflammation has been demonstrated [59, 60]. The increase in creatinine levels and the decrease in protein levels observed would be due on the one hand to damage orchestrated by carrageenan in the hepatic tissue, and on the other hand, to the necrosis of the renal cells leading to a defect in glomerular filtration [61]. The significant decrease in serum levels of ALT and AST enzymes in animals treated with doses of 125 and 250 mg/kg confirms the antiproliferative activity of the hydroethanolic extract of *V. guineensis,* and this extract contains compounds capable of influencing the formation of polypeptide-kinins during inflammation and/or to decrease the synthesis of mucopolysaccharides. The phytochemical tests carried out by Donkeng Donfack et al. [25] on the roots of this plant revealed the presence of steroids, flavonoids, and triterpenes. Previous studies have shown that many steroids and flavonoids isolated from plants [62], as well as many terpenoids [63, 64], have anti-inflammatory properties. The presence of these classes of compounds in the hydroethanolic extract of *V. guineensis* could be at the origin of the observed anti-inflammatory effects.

The results obtained show on the hematological level that the animals of the negative control group presented a significant drop in the levels of red blood cells and hemoglobin, thus suggesting signs of anemia which could be the consequence of an overproduction of the necrosis factor. Tumor-*α* (TNF-*α*) and interleukin-1 (IL-1*β*) are erythropoietin antagonists [65, 66]. Similarly, the increase in the number of white blood cells, lymphocytes, and granulocytes in the negative control could be due to stimulation of the immune system against invading antigens [67]. It could also be explained by a release of interleukins that increase the production of granulocytes and macrophage colony-stimulating factors [68]. Treatment with hydroethanolic extract of the roots of *V. guineensis* showed antianaemic (increase in red blood cell and hemoglobin levels) and immunomodulatory activities by decreasing the number of white blood cells and leukocyte species.

In order to evaluate the antiarthritic activity of the hydro-ethanolic extract of the roots of *V. guineensis*, the joint inflammation model induced by carrageenan was used. Arthritis is a chronic, inflammatory disease, systemic that causes many joints in the body to become tender and swollen, resulting in the joints becoming stiff and very painful. In the case of this study, to investigate the degree of edema caused by the local induction of arthritic inflammation, the variation in paw thickness was measured for 24 h and then for 10 days [69]. Carrageenan is generally used to induce various types of inflammation, paw edema, and acute monoarthritis in numerous animal studies. It induces inflammation in rodents, which is widely accepted as a suitable model to examine the efficacy of anti-inflammatory drugs. Arthritic edema caused by the injection of carrageenan generally results from the local action of several inflammatory mediators (prostaglandins, nitric oxide, bradykinin, 5-hydroxytryptamine, and histamine [70, 71]. The ability of the hydroethanolic extract of V. guineensis to inhibit carrageenan-induced ankle edema in animals was evaluated as a single treatment over 24 hours and as a continuous treatment for 10 days. The results clearly show that the extract significantly inhibits joint edema in a single treatment for 24 hours and continuous treatment for 10 days. These results are confirmed by the histological sections carried out in the animals which show that the negative control animals have a joint with a wide articular space, bone, and cartilage erosion, while those that received the treatment with high doses of the extract have a joint with joint erosion. These results suggest the antiarthritic properties of the hydroethanolic extract of the roots of *Vernonia guineensis*. The work of Georgewill and Georgewill [72] has shown the antiarthritic properties of *Vernonia amygdalina*, a plant from the same family as *V. guineensis*. Similarly, several flavonoids and polyphenols have anti-inflammatory and antiarthritic properties [73].

Oxidative stress is a major risk factor for many human conditions, including inflammation (acute and/or chronic) [74]. Carrageenan causes the release of some important proinflammatory cytokines (tumor necrosis factor *α* and interleukin-1*β*) which activate neutrophil infiltration [75]. It is also known that free radicals play an important role in the inflammatory (acute and chronic) response induced by carrageenan [76]. The antioxidant efficacy of the extract was determined by evaluating biomarkers of oxidative stress (liver, kidneys, brain, and marrow) of rats 10 days after intra-articular injection of carrageenan. The results show that the hydroethanolic extract of *V. guineensis* significantly increases the activities of GSH, catalase, and SOD, then significantly decreases the levels of NO and MDA in treated animals compared to untreated animals. These results suggest that this extract has antioxidant and anti-inflammatory properties that could be attributed to the presence of numerous secondary metabolites. The improvement of endogenous antioxidant status by the extract suggests that it could be very effective against oxidative stress associated with inflammatory arthritis due to its anti-inflammatory properties. This is justified by the fact that Erasto et al. [77] showed the antioxidant properties of *Vernonia amygdalina*, and that Romero-Estrada et al. [78] showed the anti-inflammatory and antioxidant properties of many flavonoids and polyphenols isolated from medicinal plants.

The anti-infammatory properties *in vitro* (COX, 5-LOX, protein denaturation, proteinase, and membrane stabilisation) and *in vivo* (formalin and carrageenan) of the hydroethanolic extract of *V. guineensis* were demonstrated in this study. The results of this study suggest that the hydroethanolic extract of *V. guineensis* is able to treat and prevent the development of inflammation (acute and chronic) and to reduce the systemic, hematological, and histological adverse effects associated with carrageenan injection. This demonstrates that the roots of *V. guineensis* extracted with water/ethanol mixture are a promising treatment for the management of acute and chronic inflammation.

## Figures and Tables

**Table 1:** Quantitative phytochemical screening of hydroethanolic extract of *V. guineensis*.

	Flavonoids (mg/g quercetin)	Phenols (mg/g catechin)	Tannins (mg/g tannic acid)
Extract	138.31 ± 1.02	223.21 ± 3.21	119.29 ± 2.32

The levels of flavonoids, total phenols, and tannins are expressed as milligram equivalents of quercetin, milligram equivalents of catechin, and milligram equivalents of tannic acid, respectively.

**Table 2:** Effect of hydroethanolic extract of *V. guineensis* on cyclooxygenase, 5-lipoxygenase, protein denaturation, proteanase, and membrane stabilisation.

	Concentration (*µ*g/ml)	Inhibition (%)				
		COX	5-LOX	Protein denaturation	Proteinase	Membrane stabilisation
Iburprofen	50	51.90 ± 1.29	43.02 ± 1.90	—	—	—
	100	63.10 ± 1.21	56.21 ± 0.76	—	—	—
	200	78.11 ± 0.33	66.34 ± 1.19	—	—	—
	400	82.21 ± 0.22	73.71 ± 1.01	—	—	—

Diclofenac	50	—	—	40.22 ± 0.89	52.55 ± 1.23	56.32 ± 1.34
	100	—	—	59.01 ± 1.21	68.03 ± 1.09	67.62 ± 0.29
	200	—	—	70. 32 ± 0.23	75.21 ± 1.92	73.01 ± 1.89
	400	—	—	83.88 ± 2.01	81.02 ± 1.12	83.90 ± 1.11

Hydroethanolic extract	50	50.10 ± 0.32	35.33 ± 0.21	44.51 ± 1.34	49.90 ± 1.22	46.52 ± 0.27
	100	62.19 ± 0.82	41.23 ± 0.39	59.20 ± 1.10	61.11 ± 2.01	59.99 ± 0.26
	200	73.29 ± 0.19	53.22 ± 0.98	67.01 ± 1.34	70.71 ± 1.01	73.27 ± 1.30
	400	80.19 ± 0.13	64.66 ± 1.11	79.02 ± 0.81	83. 88 ± 1.11	80.57 ± 1.02

The percentage values were obtained using various concentrations of test compounds and readings are presented as the mean of triplicates.

**Table 3:** General appearance and behavioral observations of acute toxicity study after hydroethanolic extract of *V. guineensis* administration.

Observation	After 4 hours				After 7 days		
	Distilled water	250 mg/kg	2500 mg/kg	5000 mg/kg	250 mg/kg	2500 mg/kg	5000 mg/kg
Alertness	Normal	Normal	Normal	Normal	Normal	Normal	Normal
Body weight	Normal	Not change	Not change	Not change	Not change	Not change	Not change
Grooming	Absent	Absent	Absent	Absent	Absent	Absent	Absent
Temperature	Normal	Normal	Normal	Normal	Normal	Normal	Normal
Restlessness	Absent	Absent	Absent	Absent	Absent	Absent	Absent
Touch response	Normal	Normal	Normal	Normal	Normal	Normal	Normal
Food intake	Normal	Normal	Normal	Normal	Normal	Normal	Normal
Pain response	Normal	Normal	Normal	Normal	Normal	Normal	Normal
Urination	Normal	Normal	Normal	Normal	Normal	Normal	Normal
Righting reflux	Normal	Normal	Normal	Normal	Normal	Normal	Normal
Pinna reflex	Present	Present	Present	Present	Present	Present	Present
Writhing	Absent	Absent	Absent	Absent	Absent	Absent	Absent
Tremors	Absent	Absent	Absent	Absent	Absent	Absent	Absent
Salivation	Normal	Normal	Normal	Normal	Normal	Normal	Normal
Convulsion	Absent	Absent	Absent	Absent	Absent	Absent	Absent
Corneal reflex	Present	Present	Present	Present	Present	Present	Present
Lacrimation	Normal	Normal	Normal	Normal	Normal	Normal	Normal
Water intake	Normal	Normal	Normal	Normal	Normal	Normal	Normal
Rate of respiration	Normal	Normal	Normal	Normal	Normal	Normal	Normal
Gripping	Normal	Normal	Normal	Normal	Normal	Normal	Normal
Skin color	Normal	Normal	Normal	Normal	Normal	Normal	Normal
Drowsiness	Not present	Not present	Not present	Present	Not present	Not present	Not present
Pupils	Normal	Normal	Normal	Normal	Normal	Normal	Normal
Sedation	No effect	No effect	No effect	Observed	No effect	No effect	No effect
Eye color	No effect	No effect	No effect	No effect	No effect	No effect	No effect
Diarrhea	Not present	Not present	Not present	Not present	Not present	Not present	Not present
General physique	Normal	Normal	Normal	Lethargy	Normal	Normal	Normal
Mortality	Nil	Nil	Nil	Nil	Nil	Nil	Nil

**Table 4:** Effect of the hydroethanolic extract of *V. guineensis* on hematology.

Treatment	Dose (mg/kg)	White blood cells (10^3^/*µ*l)	Red blood cells (10^6^/*µ*L)	Hemoglobins (g/dl)	Hematocrit (%)	Platelets (10^9^/L)
Normal control	—	5.64 ± 0.44	5.50 ± 0.31	13.90 ± 0.36	50.15 ± 4.45	292.40 ± 29.74
Negative control	—	11.46 ± 0.69^*λ*^	3.25 ± 0.15^*λ*^	10.14 ± 0.31^*λ*^	43.72 ± 0.93	394.20 ± 11.28^*β*^
Diclofenac	5	9.64 ± 0.55^*λ*^	3.42 ± 0.24^*λ*^	11.56 ± 0.48^*α*^	46.14 ± 1.62	328.80 ± 9.62

HEE	62.5	8.66 ± 0.30^*λ*b^	3.63 ± 0.22^*λ*^	9.44 ± 0.68^*λ*^	37.24 ± 1.79^*β*^	372.40 ± 10.30^*α*^
	125	7.70 ± 0.36^c^	4.37 ± 0.11^*α*a^	11.92 ± 0.67	42.56 ± 2.56	328.00 ± 14.08
	250	5.88 ± 0.12^c^	5.29 ± 0.18^c^	13.68 ± 0.35^c^	50.12 ± 0.92	298.80 ± 22.86^a^

Each value represents the mean ± standard error on the mean of 5 animals and analyzed by one-way ANOVA followed by Tukey's post test. ^a^*p* < 0.05, ^b^*p* < 0.01, and ^c^*p* < 0.001 compared with the negative control and ^*α*^*p* < 0.05, ^*β*^*p* < 0.01, and ^*λ*^*p* < 0.001 compared with the neutral control.

**Table 5:** Effect of the hydroethanolic extract of *V. guineensis* on some serum parameters.

Treatment	Dose (mg/kg)	ALT (*μ*/I)	AST (*μ*/I)	ALP (*μ*/I)	Creatinine (mg/ml)	Protein (g/dl)
Normal control	—	119.30 ± 2.86	421.90 ± 18.65	226.80 ± 2.70	0.38 ± 0.03	6.76 ± 0.51
Negative control	—	205.10 ± 7.04^*λ*^	464.70 ± 11.97	354.20 ± 5.79^*λ*^	0.43 ± 0.08	3.76 ± 0.24^*λ*^
Diclofenac	5	156.00 ± 5.37^*β*c^	455.50 ± 12.15	328.10 ± 4.68^*β*^	0.41 ± 0.06	5.31 ± 0.23

Hydroethanolic extract	62.5	207.60 ± 8.47^*λ*^	462.40 ± 14.23	346.50 ± 15.33^*λ*^	0.43 ± 0.05	3.96 ± 0.16^*β*^
	125	150.00 ± 8.91^*α*c^	454.30 ± 13.77	268.30 ± 25.81^a^	0.39 ± 0.03	5.94 ± 0.33^b^
	250	134.30 ± 3.46^c^	439.20 ± 24.98	244.00 ± 5.63^b^	0.38 ± 0.03	6.70 ± 0.27^b^

Each value represents the mean ± SEM of 5 animals. ALP: alkaline phosphatase; AST: aminotransferase; ALT: alanine aminotransferase. ^a^*p* < 0.05, ^b^*p* < 0.01, and ^c^*p* < 0.001 are statistically significant compared to negative control; ^*α*^*p* < 0.05, ^*β*^*p* < 0.01, and ^*λ*^*p* < 0.001 are statistically significant compared to the neutral control.

## Data Availability

All data generated or analyzed during this study are included in this published article.

## Additional Points

*Plant Authentication*. The plants used in this study were authenticated at the National Herbarium of Yaoundé, Cameroon, under the name of LETOUZEY under the number 4869/SRFK.
